# Genomic signatures of relaxed disruptive selection associated with speciation reversal in whitefish

**DOI:** 10.1186/1471-2148-13-108

**Published:** 2013-05-30

**Authors:** Alan G Hudson, Pascal Vonlanthen, Etienne Bezault, Ole Seehausen

**Affiliations:** 1 Division of Aquatic Ecology & Macroevolution, Institute of Ecology & Evolution, University of Bern, Baltzerstrasse 6, Bern, CH, 3012, Switzerland; 2 Department of Fish Ecology & Evolution, EAWAG Centre of Ecology, Evolution and Biogeochemistry, Seestrasse 79, Kastanienbaum, CH, 6047, Switzerland; 3 Department of Ecology & Environmental Science, Umeå University, Umeå, SE, 901 87, Sweden; 4 Department of Biology, Reed College, 3203 SE Woodstock Blvd, Portland, OR, 97202, USA

**Keywords:** *Coregonus*, Population genomics, Adaptive radiation, Speciation reversal, Relaxed selection, Heterogeneous genomic divergence, Ecological speciation, Speciation-with-gene-flow, Eutrophication, Divergence hitchhiking

## Abstract

**Background:**

Speciation reversal: the erosion of species differentiation via an increase in introgressive hybridization due to the weakening of previously divergent selection regimes, is thought to be an important, yet poorly understood, driver of biodiversity loss. Our study system, the Alpine whitefish (*Coregonus* spp*.*) species complex is a classic example of a recent postglacial adaptive radiation: forming an array of endemic lake flocks, with the independent origination of similar ecotypes among flocks. However, many of the lakes of the Alpine radiation have been seriously impacted by anthropogenic nutrient enrichment, resulting in a collapse in neutral genetic and phenotypic differentiation within the most polluted lakes. Here we investigate the effects of eutrophication on the selective forces that have shaped this radiation, using population genomics. We studied eight sympatric species assemblages belonging to five independent parallel adaptive radiations, and one species pair in secondary contact. We used AFLP markers, and applied F_ST_ outlier (BayeScan, Dfdist) and logistic regression analyses (MatSAM), to identify candidate regions for disruptive selection in the genome and their associations with adaptive traits within each lake flock. The number of outlier and adaptive trait associated loci identified per lake were then regressed against two variables (historical phosphorus concentration and contemporary oxygen concentration) representing the strength of eutrophication.

**Results:**

Whilst we identify disruptive selection candidate regions in all lake flocks, we find similar trends, across analysis methods, towards fewer disruptive selection candidate regions and fewer adaptive trait/candidate loci associations in the more polluted lakes.

**Conclusions:**

Weakened disruptive selection and a concomitant breakdown in reproductive isolating mechanisms in more polluted lakes has lead to increased gene flow between coexisting Alpine whitefish species. We hypothesize that the resulting higher rates of interspecific recombination reduce either the number or extent of genomic islands of divergence surrounding loci evolving under disruptive natural selection. This produces the negative trend seen in the number of selection candidate loci recovered during genome scans of whitefish species flocks, with increasing levels of anthropogenic eutrophication: as the likelihood decreases that AFLP restriction sites will fall within regions of heightened genomic divergence and therefore be classified as F_ST_ outlier loci. This study explores for the first time the potential effects of human-mediated relaxation of disruptive selection on heterogeneous genomic divergence between coexisting species.

## Background

Understanding the evolutionary processes that control the diversification, coexistence and persistence of species has taken on a heightened urgency as human-induced environmental changes and stressors have increased in prevalence and the augurs of a new, anthropogenic mass extinction become more evident [1-3]. Changes in abiotic and biotic environmental conditions will alter the direction, strength and form of selection that generates and maintains species differences and species coexistence, particularly among recently diverged species. How populations of such species respond to environmental change will depend on the severity, rate and duration of the change and the associated selection pressures experienced. Negative population growth is just one way species can go extinct; another scenario for species loss in the face of environmental change is speciation reversal [4]. Here, a reduction in environmental heterogeneity increases gene-flow and/or weakens disruptive selection, resulting in a break down in pre-zygotic and/or extrinsic post-zygotic reproductive isolation: leading to the fusion of previously distinct species through introgressive hybridization [5-8].

The effects of human-induced habitat alteration on the levels of intra- and interspecific diversity within adaptive radiations have generally been studied through the measurement of changes in the frequencies and distributions of neutral genetic markers and/or putative adaptive trait values among and within the affected species. Examples from vertebrate radiations indicate a weakening of disruptive selection during the course of environmental perturbations and concomitantly an increase in hybridization between coexisting species, as the distribution of feeding resources and/or habitats for breeding becomes homogenized and prezygotic and extrinsic postzygotic reproductive isolation weakens. This process is accompanied by the complete or near complete fusion of previously discrete gene pools into single, albeit genetically diverse populations, and trait distributions losing multimodality [5,6,9].

During ecological speciation gene flow between diverging populations is expected to be restricted mainly at loci underpinning traits involved in divergent adaptation and areas of the genome in physical linkage with these loci; so called “genomic islands of divergence” [10,11]. Among recently diverged species, the balance between disruptive selection and homogenizing gene flow will determine the extent of genetic differentiation achieved and its genomic distribution [12,13]. This heterogeneous genomic divergence will be at its most extreme during moderate to high gene flow and strong exogenous disruptive selection, predicted to characterize the early stages of sympatric/parapatric speciation scenarios [14,15]. As speciation progresses, genomic islands may increase both in number and extent, as inter-specific recombination rates decrease, potentially enhancing reproductive isolation via divergence hitchhiking (DH) [10,13,16]. At later stages of ecological speciation, as many loci diverge among incipient species, genome-wide effective gene flow will be reduced even at unlinked loci: divergence hitchhiking being superseded by genome hitchhiking (GH) [16]. Many studies have employed population genomic methods to identify genomic regions with unusually strong differentiation as holding potential candidate genes for disruptive selection between diverging taxa *e*.*g*. [17-21]. However little effort has been devoted to investigating how changes in natural selection regimes through anthropogenic perturbation affect the occurrence and size of genomic islands of divergence between diverging (now converging) species, and nothing is published about this to our knowledge.

The Alpine whitefish (*Coregonus* spp.) radiation is native to around 40 lakes in the headwaters of three major European river drainages: the Rhine, Rhône and Danube [22]. Colonising following the glacial retreat (15,000 – 20,000 years BP), this monophyletic group has diverged in-situ into at least seven species flocks, centred on single large lakes or previously connected sister lakes [23-25]. The constituent species of these lake or super-lake flocks are generally each other’s closest relatives, indicating intralacustrine speciation [25]. Historically, *Coregonus* species richness varied from one to six endemic species per lake. Coexisting species differ in traits linked to feeding and reproductive ecology, with very similar ecotypes having evolved repeatedly in several lakes [23,25,26].

During the 20th Century, many of the lakes in which the Alpine *Coregonus* radiation occurs have seen unprecedented anthropogenic impacts. Run-off from agriculture and insufficient sewage treatment massively increased the nutrient input into many lakes, leading to eutrophication of these naturally oligotrophic lakes. The increase in biologically available phosphorus had several effects on lake ecology, changing the community structure of phytoplankton and their zooplankton grazers, therefore affecting the prey resource base for whitefish species [8,27]. Increased productivity also resulted in increased bacterial decomposition, reducing levels of available oxygen in the hypolimnion [28,29]. This de-oxygenation is most severe at the sediment-water interface, where whitefish egg development occurs [29]. A consequence of this is the reduction of the available depth gradient partitioned between whitefish species during spawning and concomitantly an increase in rates of hybridization between shallow and deep spawning species [8,30,31]. In more extreme cases de-oxygenation renders deeper areas of the lake anoxic, potentially reducing the available area and diversity of foraging habitat [29]. During this period of unprecedented environmental change, the Alpine whitefish radiation experienced a loss of around 38% of its pre-eutrophication species richness [8]. This diversity loss appears to have been driven by a combination of negative population growth combined with the genetic collapse of coexisting species through speciation reversal. Indeed by comparing historical population samples taken before large-scale eutrophication with samples taken from the same species post-eutrophication, we see a clear signal of lower levels of neutral genetic and phenotypic differentiation between coexisting species within lakes that had high levels of eutrophication, in comparison to species from lakes that have never been strongly eutrophic [8].

Here we present the first genome scan analysis that explores the impact of anthropogenic environmental change on heterogeneous genomic divergence in an adaptive radiation. We studied whitefish species from nine Alpine lakes, comprising five phylogenetically independent *Coregonus* adaptive radiations and one species assemblage arising from recent anthropogenic secondary contact. We employ genome scans of 183 individuals comprising 851 AFLP markers. We subject these AFLP loci to F_ST_ outlier detection to identify candidate regions in the genome that likely evolved under disruptive selection, and logistic regression analyses methods to link candidate regions to adaptive traits that they potentially influence. For all analysis methods, we consistently find a trend for more outlier and more trait-associated loci in those lakes that experienced lower levels of eutrophication. Here we provide the first genetic evidence for the weakening of the genomic signature of disruptive selection and divergent adaptation that are predicted to cause speciation reversal following human-induced habitat modification.

## Methods

### Study system & sampling

**Figure 1:**
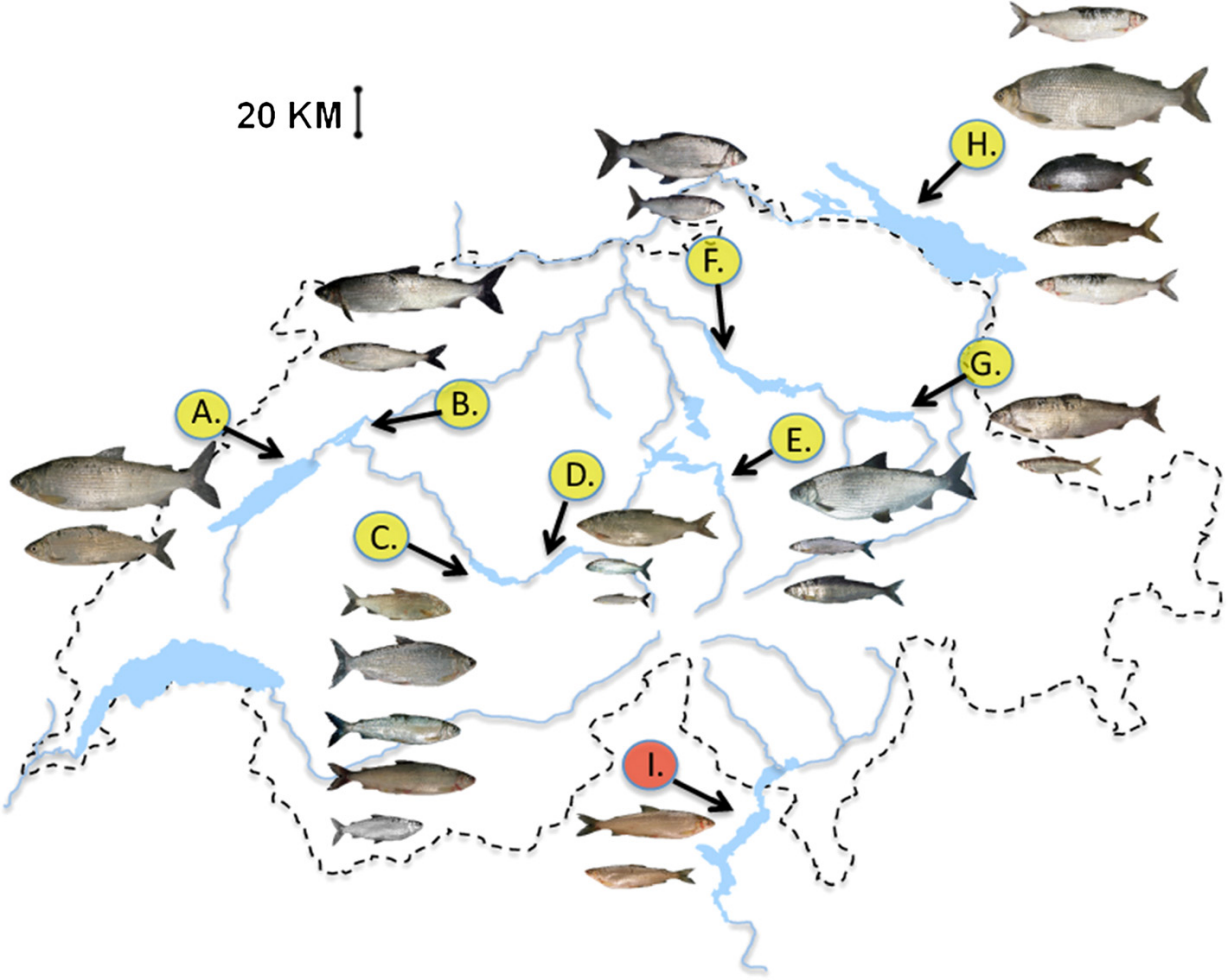
**Geographic locations of the species flocks studied within Switzerland.** Map showing the number of species included, their sampling location and their representative phenotypes (species names listed from above to below). (**A**) Lake Neuchâtel: (*C*. *palaea*, *C*. *candidus*), (**B**) Lake Biel: (*C*. *palaea*, *C*. *confusus*), (**C**) Lake Thun: (*C*. *alpinus*, *C*. *sp*. “balchen”, *C*. *fatioi*, *C*. *sp*. “felchen”, *C*. *albellus*), (**D**) Lake Brienz: (*C*. *sp*. “balchen”, *C*. *sp*. “felchen”, *C*. *albellus*), (**E**) Lake Lucerne: (*C*. *sp*. “bodenbalchen”, *C*. *zugensis*, *C*. *nobilis*), (**F**) Lake Zuerich: (*C*. *duplex*, *C*. *heglingus*), (**G**) Lake Walen: (*C*. *duplex*, *C*. *heglingus*), (**H**) Lake Constance: (*C*. *sp*. “weissfelchen”, *C*. *arenicolus*, *C*. *sp*. “Alpenrhein”, *C*. *macrophthalmus*, *C*. *wartmanni*), (**I**) Lake Maggiore: (*C*. *sp*. “lavarello”, *C*. *sp*. “bondella”). Yellow circles denote native species flocks. Red circle denotes anthropogenic origin and secondary contact.

We sampled *Coregonus* species from nine different lakes, each containing between two and five coexisting species. Individuals were collected between 2004 and 2006 on their respective spawning grounds using overnight gill-netting. Information about the species and lakes sampled can be found in Table 1 and Figure 1. Species were assigned to feeding ecotype using their mean gill-raker number, a correlate of feeding ecotype [32]: very low gill-raker numbers (VLGR <25 gill-rakers), low (LGR 25–30), medium (MGR 31–35) and high (HGR >35). We used both the historical maximum total dissolved phosphorus concentration (P_max_ (P_tot_) (μg l^-1^)) and contemporary minimum oxygen concentration at depth (Min. O_2_ (mg l^-1^)) as indicators of the intensity of anthropogenic nutrient pollution: both variables potentially show different aspects of the speciation reversal process, relating to the duration and intensity of habitat disruption and its effects on genomic divergence. P_max_ was used as a surrogate for the maximum level of pollution experienced by each lake system and therefore to measure the impact on interspecific genomic divergence when pollution was at its most severe. Min. O_2_ was used to gauge the extent to which lake systems have currently returned to their pre-eutrophication state, and therefore represents the potential ongoing effects of relaxed selection on genomic divergence. Both P_max_ and Min. O_2_ have been shown to strongly correlate (positively and negatively respectively) with a loss in intralacustrine *Coregonus* species diversity [8].

**Table 1:** Geographical location, lake surface area, lake trophic state, taxonomy, spawning/feeding ecotype designation and the number of individuals of each species included, for all species flocks analysed

**Lake**	**Co-ordinates**	**Surface Area (km**^**2**^**)**	**P**_**max **_**(P**_**tot**_**) (μg l**^**-1**^**)**	**Min. O**_**2 **_**(mg l**^**-1**^**)**	**Species**	**Ecotype**	**# Individuals**
Neuchâtel	46°53 N;	217.9	50	3.2	*C. palaea*	LGR, W	7
	6°5 E				*C. candidus*	MGR, W	7
							**14**
Biel	47°04 N;	39.3	132	0.5	*C. palaea*	LGR, W	6
	7°1 E				*C. confusus*	MGR, W	7
							**13**
Thun	46°41 N;	48.4	21	7.6	*C. alpinus*	VLGR, S	7
	7°42 E				*C. sp.* “balchen”	LGR, W	6
					*C. fatioi*	MGR, W	8
					*C. sp.* “felchen”	MGR, W (Deep)	7
					*C. albellus*	HGR, S	7
							**35**
Brienz	46°43 N;	29.8	17	9.3	*C. sp.* “balchen”	LGR, W	4
	7°58 E				*C. sp.* “felchen”	MGR, W	7
					*C. albellus*	HGR, S	5
							**16**
Lucerne	47°01 N;	114	34	3.4	*C. sp.* “bodenbalchen”	LGR, W	10
	8°22 E				*C. zugensis*	HGR, S/W	9
					*C. nobilis*	HGR, S	7
							**26**
Constance	47°36 N;	539	87	6.0	*C. sp.* “weissfelchen”	LGR, W	7
	9°26 E				*C. arenicolus*	LGR, W	6
					*C. sp.* “Alpenrhein”	MGR, W	5
					*C. macrophthalmus*	HGR, W	6
					*C. wartmanni*	HGR, W (Pelagic)	6
							**30**
Walen	47°07 N;	24.1	26	6.1	*C. duplex*	LGR, W	8
	9°12 E				*C. heglingus*	HGR, S/W	9
							**17**
Zuerich	47°13 N;	88.4	119	1.1	*C. duplex*	LGR, W	11
	8.43 E				*C. heglingus*	HGR, W	7
							**18**
Maggiore	45°58 N;	212.5	37	n/a	*C. sp.* “lavarello”	MGR, W	8
	8°4 E				C. *sp*. “bondella”	HGR, W	6
							**14**

Feeding ecotype: VLGR = Very low mean gill-raker number (<25 gill-rakers), LGR = low (25–30), MGR = medium (31–35) and HGR = high (>35). Reproductive ecotype: W = winter spawner (October-January), S = summer spawner (July-September). P_max_ = peak historical levels of phosphorus enrichment. Min. O_2_ = contemporary minimum oxygen concentration at depth.

Lakes Neuchâtel and Biel are connected by *ca*. 8 Km of river and their three endemic *Coregonus* species form part of a monophyletic group with other nearby single-species lakes [23,25]. The five *Coregonus* species inhabiting connected (*ca*. 5 km of river) lakes Thun and Brienz, form a monophyletic clade too, except *C*. *fatioi*, which appears to have had some genetic input from Lake Constance *Coregonus* species [23,25]. The endemic species flock of Lake Lucerne consists of potentially five coexisting species [Lundsgaard *et al*. unpublished observations, 26]. For this study no samples of two recently discovered forms, intermediate in size and gill-raker counts, were available. Lake Constance consists of two major basins, Obersee (476 Km^2^) and Untersee (63 Km^2^). Previously with up to six reported species, heavy eutrophication has lead to the extinction of at least one species, the VLGR *C*. *gutturosus*[8,22]. Four species coexist in the Obersee, with the *C*. *sp*. “weissfelchen” found in the Untersee basin [23,25]. Lakes Walen and Zuerich are connected by *ca*. 17 Km of river, the two *Coregonus* species endemic to these lakes, together with whitefish from two nearby, single-species lakes, form an independent, monophyletic radiation. Additionally from Walen and Zuerich, one or two poorly known species, may also have become extinct [8,26]. The two coexisting whitefish species of Lake Maggiore are introduced; *C*. *sp*. “lavarello” is most closely related to *C*. *sp*. “Zugerbalchen” from Lake Zug [23,25], while *C*. *sp*. “bondella”, groups closest to *C*. *zugensis* from Lake Lucerne [25]. This species pair represents a case of recent secondary contact, *C. sp*. “lavarello” and *C*. *sp*. “bondella” being introduced *ca*. 1860 and 1950 respectively [33].

### AFLP analysis

Total genomic DNA was extracted using the Wizard Genomic DNA purification kit (Promega, Madison, WI). A genome scan of putatively independent loci was then carried out using AFLPs, following a protocol modified from [34], with combined restriction/ligation steps, using *Eco*RI and *Mse*I restriction enzymes. Selective PCRs were performed with 14 different primer-pairs (ACG-CAC, ACT-CAC, ACC-CAG, ACG-CAG, ACT-CAG, ACT-CAT, ACA-CGC, ACC-CGC, ACT-CGC, ACT-CGG, ATG-CTC, ATG-CTG, AAG-CTT, ACG-CTT). Fragment separation was carried out by electrophoresis on a Ceq-8000 automatic sequencer (Beckman-Coulter). AFLP traces were then screened and scored for peak presence/absence using GeneMarker 1.85 (SoftGenetics, LLC, State College, PA). Individual traces were automatically scored and then bins were visually inspected and manually adjusted. Only AFLP loci between 55–400 base pairs were scored. To check levels of marker reproducibility 30 individuals were repeated (16% of the total analysed samples). The level of reproducibility per locus was then calculated for each of the 14 genotyped primer-pairs [35].

### Genomic outlier analysis

**Figure 2:**
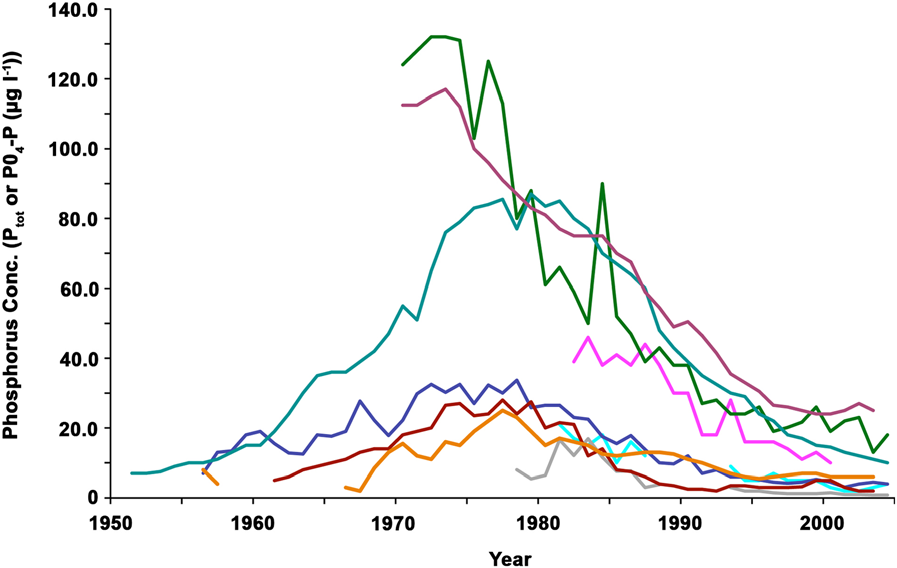
**Yearly trends in total phosphorus concentration (μg l**^**-1**^**) for sampled lakes of the Alpine *Coregonus *radiation.** Phosphorus measurements were taken during lake overturning (modified from [8]). Colours of trend lines refer to lake identity: Neuchâtel (pink), Biel (green), Thun (light blue), Brienz (red), Lucerne (dark blue), Constance (purple), Walen (grey), Zuerich (black) and Maggiore (orange).

All outlier analyses and logistic regressions were run on the nine lake flock data sets separately. This avoided potential violations of the demographic models employed in the outlier detection methods that could be caused by hierarchical structuring of genetic variation [36,37], present in the Alpine whitefish radiation [25]. Additionally, with this analysis design, we were able to compare paired lake flock data sets with similar evolutionary histories and patterns of ecological diversification, but differing in their nutrient enrichment history: Lake Walen (very low level of nutrient enrichment) *vs.* Lake Zuerich (high), Lake Biel (high) *vs.* Lake Neuchâtel (moderate to high), and Lake Thun (low) *vs.* Lake Brienz (very low) (Figure 2).

Two different F_ST_-based approaches to search for selection candidate loci, suitable for dominant markers were used: Dfdist_d[38] and BayeScan[39]. Dfdist estimates the probability of a locus to be under selection according to its observed F_ST_ and He, compared to simulated neutral distributions computed for an average neutral F_ST_ observed in the empirical data set (the trimmed mean F_ST_), effective population size and minimum levels of polymorphism [38]. In contrast, BayeScan directly estimates the probability of each locus being subject to selection by comparing the likelihoods of neutral and selective models [39]. As the power to detect stabilising selection will be low among recently diverged species, only candidate loci showing unusually high levels of genetic differentiation, implicating disruptive natural selection, were counted [38]. F_ST_ outlier analyses were first run on each multi-species lake data set (global comparisons among all species sampled in a lake), followed by all possible pairwise comparisons between individual species for lakes with more than two species.

In the Dfdist analysis the mean F_ST,_ used for simulating a null distribution of F_ST_ values for loci evolving under neutrality, was estimated using a trimmed mean of 0.3 [38]. The null distribution was generated using 50,000 simulated loci. In the empirical AFLP data set the maximum allele frequency was set to 0.98. AFLP loci with F_ST_ values above the upper 95th quantile of the null distribution were considered candidate loci for being under disruptive natural selection.

In the BayeScan analysis, model parameter estimation was automatically tuned during the pilot runs (ten runs, length 2,000). A 10,000 iteration burn-in was found to be sufficient for convergence between MCMC chains. Sample sizes of 10,000 and a thinning ratio of 50 were used as suggested by [39]. Prior to the analysis all non-polymorphic loci (fixed presence or absence alleles) were removed as this could bias the analysis (M. Foll pers. comm.). Loci were considered as candidates for being under divergent selection if their log_10_ Bayes Factor scores were ≥ 0.5 (“Substantial” on Jeffrey’s scale of evidence) [39].

To understand the effects of loci under selection on the phylogenetic relationships within the Alpine whitefish radiation, three different sets of Neighbour-joining (NJ) population-based trees were created: (1) using all genotyped AFLP loci (835 loci), (2) a subset comprising only outlier loci identified by Dfdist and BayeScan in either global or pairwise comparisons (96 loci), (3) a subset comprising only neutral loci remaining following removal of outlier loci (739 loci). Nei’s genetic distances between populations were calculated in AFLP-SURV 1.0 [40]. NJ trees were reconstructed from these distances using the program Neighbor in the software package PHYLIP 3.67, with 1,000 bootstrap replicates [41]. To quantify the levels of parallel ecotypic trends among lakes in the different loci subsets, following Allender *et al*. [42], Mantel randomizations were performed for neutral and outlier loci subsets for a matrix of Nei’s genetic distances and a design matrix encoding feeding ecotype. In the design matrix, species belonging to the same ecotype were encoded D = 0, D = 1 encoding assignment to different ecotypes. As the Maggiore species are non-native, they were excluded from this analysis. Mantel randomizations of 10,000 permutations were carried out in Arlequin 3.5 [43].

To assess the statistical independence of individual outlier loci from each other, levels of linkage disequilibrium (LD) were estimated for different AFLP loci subsets. For each species, genotyped AFLP loci were split into exclusive subsets: neutral and outlier. The outlier subsets comprised all loci identified as outliers by Dfdist analysis, during global-level comparisons within their respective species flock. LD $\left({\bar{r}}_{d}\right)$ values were calculated in the program Multilocus 1.3 [44]. Pairwise ${\bar{r}}_{d}$ values were calculated for all possible pairs of loci within each neutral and outlier loci subset, for each species. The significance of differences in mean pairwise LD between neutral and outlier loci subsets, across populations, was tested using paired *t*-tests.

### Divergent trait measurement

The first two trait categories measured, spawning depth and time, are related to reproductive ecology [26,31]. (1) Spawning date influences the time of emergence of the fry and therefore may influence survival through different mechanisms such as food availability, predation or over-wintering mortality [45-48] and has been show to transgressively segregate in whitefish hybrids [48,49]. Importantly, spawning date also influences temporal reproductive isolation between species [26]. The spawning date for each individual was taken as the day of capture on spawning site, adjusted so that the earliest caught fish (summer spawning) were caught on day 1, therefore those fish caught *x* days after the first fish were given the spawning date of *x*. Fish caught on the same date of different calendar years would be given the same spawning date. (2) Spawning depth is likely related to physiological or behavioural adaptations to depth [50], to different predation pressures [51], or to differences in egg size and development [48]. Also spawning depth influences spatial reproductive isolation between species [8,26,31]. Spawning depth for each individual was estimated by finding the average depth over which the gill nets were set during each sampling event.

**Figure 3:**
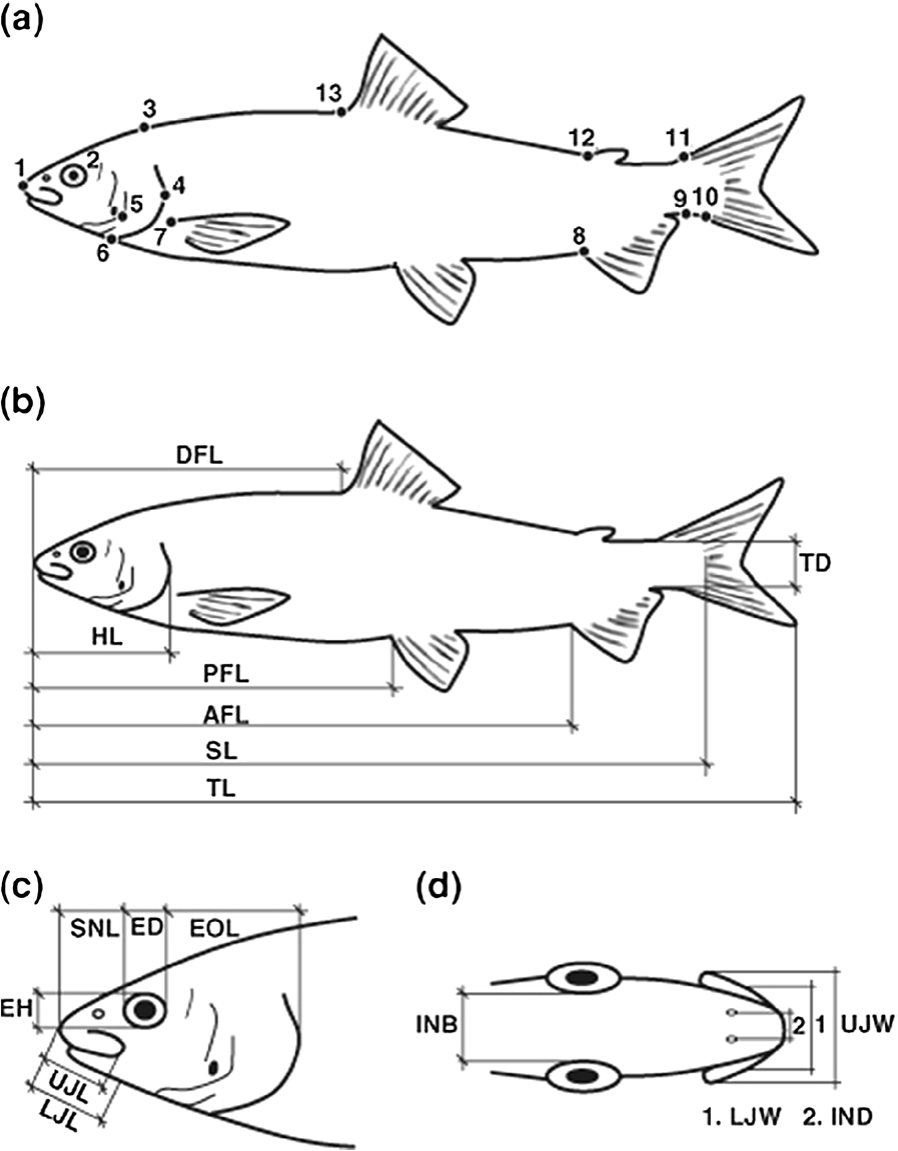
**Landmarks and distance-based measurements used in the analysis of *Coregonus *body shape.** 13 landmarks used to analyse body shape with geometric morphometrics (**a**). Fifteen linear morphometric measurements taken for each individual: (DFL) length to dorsal fin, (PFL) length to pelvic fin, (AFL) length to anal fin, (TD) tail depth, (HL) head length, (ED) eye diameter, (SNL) snout length, (EOL) eye to operculum length, (EH) eye height, (LJL) lower jaw length, (UJL) upper jaw length, (INB) intra-orbital distance, (IND) intra-nostril distance, (LJW) lower jaw width, (UJW) upper jaw width (**b-d**). Modified from [34].

Finally we measured three trait categories primarily related to feeding ecology: gill-raker number, linear morphometric distances and geometric shape analyses. (3) Gill-raker number has been shown to be correlated with feeding niche in Scandinavian whitefish [52] and to have a high heritability [32]. Gill-raker numbers were counted on the first gill arch from the left side of each fish. (4–5) Overall morphological variation among fish was measured using (4) linear morphological distances (LM) and (5) geometric morphometrics of body shape (GM). Shape aspects are potentially targets of disruptive selection, as revealed in Q_ST_-F_ST_ comparisons in lakes of the Alpine radiation [31] and show significant phenotype-environment correlations in Scandinavian *Coregonus* species flocks [53]. (4) Fifteen different LM measurements, based on those described in Chouinard, Pigeon & Bernatchez [54], were taken using digital callipers on 1090 individuals, comprising 38 whitefish populations/species from 22 lakes in the Alpine region: to capture the major axes of morphological divergence across the radiation, (Figure 3b-d). Size correction of the measured traits was performed using common principal components analysis (CPCA), followed by Burnaby's back projection method (BBPM) through the CPCBP package [55] in R (R Development Core Team, 2004). Principal component analysis (PCA) was then performed on the transformed data in SPSS 16 (SPSS Inc., Chicago Illinois, USA). The resulting PC 1–3 scores of the AFLP-genotyped individuals from the nine lake flocks were taken for logistic regression analysis. For PC 1 (30% of variation) traits with positive loadings were mainly body length traits (AFL, PFL, DFL, HL), whereas head measures particularly jaw width traits (LJW, UJW, UJL, ED, SEL) had negative loadings. PC 2 (24%) had positive loadings for head measures (HL, ED, SEL, EH, LJL, UJL) and negative loadings for traits: AFL, PFL, DFL, TD, LJW, UJW. PC 3 (10%) had strongly positive loadings for eye shape (ED, EH) and negative loadings for: SEL, UJL, INB, SNW. (5) GM landmark-based analysis of body shape variation was carried out on digital images of 1545 fish comprising 41 populations/species from 22 lakes, using 13 homologous landmarks (Figure 3a). Digital landmark placement was carried out in tpsdig2 (http://life.bio.sunysb.edu/morph/). All further shape analysis was carried out in MorphoJ 1.02d [56]. Generalized least-squares Procrustes super-imposition was carried out on landmark configurations. Allometric size correction was then carried out by the pooling of species-specific regressions of Procrustes coordinates against individual standard length. Following PCA, PC 1 was found to be mostly artifactual (associated with dorso-ventral flexion of dead fish) and was discarded. Therefore individual PC scores for PC 2 (13%), PC 3 (9%) and PC4 (9%) were used for further analysis. Shape changes along PC 2 (13%) were associated with changes in head length. PC 3 (9%) was associated mainly with the amount of up/down rotation of the head, and changes in body depth. PC 4 (9%) was mainly associated with changes in the length of the caudal peduncle and body depth.

### Association between genomic outlier loci and putative adaptive traits

Univariate logistic regressions were carried out in the program MatSAM 2Beta [57]. This approach analyses the relationship between alleles at a given AFLP locus and specific values of an explanatory variable, such as putative adaptive traits [57]. For each AFLP locus, the genotype of individuals was regressed against the individual values for each of the five putative adaptive trait variables measured: spawning date, spawning depth, gill-raker number, PC score on principal axes of morphological variation resulting from LM measurements and GM analysis of shape. Each suite of genotype/phenotype regressions were calculated separately for each of the nine species flock data sets. Individuals with missing phenotypic data were removed from the logistic regression for that specific trait. The adaptive trait values for each individual, used in logistic regressions, can be found in Additional file 1: Table S1. The significance of the association between the frequency of an allele at a locus and the value of each trait was assessed using the *G*-statistic at the 0.01, 0.05 and 0.1 significance levels, with Bonferroni correction using the number of AFLP loci used in each regression analysis.

### Tests of association between heterogeneous genomic divergence and eutrophication

To test if the distribution over lakes of the detected genomic signal of disruptive selection (number of outlier loci and trait-associated loci) was robust to analysis method (Dfdist, BayeScan, MatSAM), correlations between the numbers of loci detected by each analysis method were carried out. To test the hypothesis that eutrophication causes a decrease in the number of identified outlier/trait-associated loci, one-tailed regressions were calculated of the number of loci detected in global comparisons within each species flock, against maximum historical phosphorus concentration (P_max_) and current minimum oxygen concentration at depth (Min. O_2_) of the lake the species flock inhabits. This test was repeated for each of the three methods employed (Dfdist, BayeScan, MatSAM).

As a comparison the effect of eutrophication on neutral markers was also tested by linear regression of P_max_ and Min. O_2_ against F_ST_ values from each lake flock calculated from three different marker sets: (1) microsatellites (maximum within-lake pairwise F_ST_ value), (2) all AFLP loci (highest within-lake weighted mean F_ST_ in Dfdist from pairwise comparisons), (3) neutral AFLP loci (highest within-lake weighted mean F_ST_ in Dfdist from pairwise comparisons, following the removal of all candidate loci identified by the Dfdist, BayeScan, MatSAM analyses). Lake flock microsatellite F_ST_ values resulting from between ten (Maggiore only) or eleven unlinked loci were taken from [8,58], Hudson *et al*. unpublished observations. We also calculated pairwise correlations between the three independent estimates of F_ST_ to see how congruent their signals were. All statistical analyses were carried out in SPSS 16 (SPSS Inc., Chicago Illinois, USA).

## Results

The total number of AFLP loci scored per lake flock varied from between 355–405 (Dfdist) and 216–306 (BayeScan) (Table 2). The number of whitefish genotyped per flock ranged from 13 to 35 (Tables 1, 2), depending largely on the number of constituent species. For species-pairwise analyses the number of loci genotyped ranged from between 334–380 (Dfdist) and 177–247 (BayeScan), and the number of individuals from 9–19. Average per locus levels of reproducibility across the fourteen primer pairs exceeded 95%. Identity codes, the total number of loci (both polymorphic and fixed) and average reproducibility per primer pair can be found in Additional file 2: Table S2.

**Table 2:** **Number of selection candidate loci identified using DFDISTand BAYESCANwithin individual lake flocks (global comparisons)**

**Species Flock**	**# Species**	**# Individuals**	**# Poly. Loci**	**# Candidate loci DFDIST**		**Total**	**# Candidate loci BAYESCAN**		**Total**	**Shared Loci**	**% Similarity**
				**≥ 0.95**	**≥ 0.99**		**≥ 0.5**	**≥ 1**			
Neuchâtel	2	14	241	5	0	5	2	0	2	2	40
Biel	2	13	262	1	1	2	0	0	0	0	0
Thun	5	35	306	18	4	22	3	1	4	4	18
Brienz	3	16	216	9	2	11	1	1	2	2	18
Lucerne	3	26	266	9	2	11	3	0	3	2	18
Constance	5	30	279	4	1	5	0	0	0	0	0
Walen	2	17	229	2	2	4	2	0	2	2	50
Zuerich	2	18	230	5	3	8	3	1	4	4	50
Maggiore	2	14	223	11	2	13	1	2	3	3	23
			**Total**	64	17	81	15	5	20		

Presented are the numbers of species, individuals and polymorphic AFLP loci comprising each species flock data set. Identified Dfdist outlier loci are grouped according to how many fell between the 95% and 99% quantiles (≥ 0.95), and how many fell above the 99% quantile (≥ 0.99). BayeScan outlier loci are grouped by their Bayesian posterior probability of being under selection (Log_10_ Bayes factor scores): “substantial” 0.5 to 1 (≥ 0.5), “strong” 1 to 1.5 (≥ 1). Also shown: the number of AFLP loci identified as selection candidates by both outlier detection methods and the overall similarity between sets of candidate loci recovered in Dfdist and in BayeScan, for each species flock.

### F_ST_ outlier scans

The Dfdist analyses detected disruptive selection candidate loci in every species flocks. The largest number of outliers was found in the Lake Thun species assemblage (22) (Table 2, Additional file 3: Table S3a). Most loci detected (64 of 81) fell within the 95–99% quantiles. Loci with F_ST_ values above the 99% quantile were found in L. Thun (four), L. Zuerich (three), L. Brienz (two), L. Lucerne (two), L. Walen (two), L. Biel (one) and L. Constance (one). Of the four loci found to be outliers in multiple lakes, three loci showed parallel allelic trends among similar ecotypes. CAGC-235 was an outlier in the Thun, Lucerne, Zuerich and Maggiore species flocks. For this locus prevalence of the presence allele was reduced in Zuerich LGR *C*. *duplex*, Lucerne LGR *C*. *sp*. “bodenbalchen”, Thun MGR *C*. *fatioi* and Maggiore MGR *C*. *sp*. “lavarello”. The presence allele of CGAG-186 was at a higher prevalence in Brienz MGR *C. sp. "*felchen*"* and Thun/Brienz HGR *C. albellus*. At TGTG-183 there was a decreased prevalence of the presence allele in LGR *C. sp. "*balchen*"* from both lakes Brienz and Thun and also in Thun MGR *C*. *fatioi*. Pairwise Dfdist analyses (Additional file 3: Table S3b) revealed 36 additional loci not recovered in the global analyses. Six of these loci were identified as outliers in several pairwise species comparisons from different lakes, with three showing parallel trends among similar ecotypes. Locus CAGC-342 had a high prevalence of the presence allele in HGR L. Brienz *C*. *albellus* and HGR L. Constance *C*. *wartmanni*. TGTG-271 showed parallel trends in HGR Lucerne *C*. *zugensis* and HGR Maggiore *C*. *sp*. “bondella”. Finally the presence allele of CGAG-355 had a reduced frequency in MGR Constance *C*. *sp.* “Alpenrhein” and MGR Maggiore *C*. *sp*. “lavarello”.

From the global lake-level BayeScan analyses, we find disruptive selection candidate loci between the constituent species in every lake except Biel and Constance (Table 2, Additional file 4: Table S4a). 15 out of the 20 outlier loci identified had Bayes factor scores of “substantial” on Jeffrey’s scale of evidence (≥ 0.5 – 1). One locus scoring “strong” (≥ 1 – 1.5) was found in each of Thun, Brienz and Zuerich flocks and two in Maggiore. Similar to the Dfdist analyses, CAGC-235, was detected as an outlier in multiple lakes. The presence allele for this locus had a low prevalence in the Thun MGR *C*. *fatioi* and Lucerne LGR *C. sp. "*bodenbalchen*"*. Pairwise BayeScan analyses (Additional file 4: Table S4b) revealed five additional outlier loci not recovered in the global analyses at lake level. None of these loci were outliers in more than one species pair.

Overall the Dfdist analysis identified over four times more outlier loci than the BayeScan analysis (81 *vs*. 20). Similarity of identity of candidate loci identified with either method (global F_ST_ analysis at lake level) was lowest in lakes with low average mean F_ST_ (for Lakes Constance and Biel zero loci were found in the BayeScan analyses, but four and two loci respectively in Dfdist analyses) and/or with more than two species coexisting (L. Brienz, L. Thun and L. Lucerne; only 18% of loci identified by both detection methods) (Table 2). Deviations were in all cases due to Dfdist detecting higher numbers of F_ST_ outliers.

Consensus trees from the three different subsets of AFLP loci showed high levels of congruence (Additional file 5: Figure S1). Compared to the all loci consensus tree, the outlier loci subset tree showed little obvious increase in ecotype clustering. Design matrices of feeding ecotype designation explained slightly more variance in genetic distances between species when the latter were calculated from the outlier loci subset rather than the neutral loci subset (*r* = 0.07 and *r* = 0.04 respectively), but none of these Mantel randomizations were significant (0.004%, *P* = 0.12 and 0.001%, *P* = 0.22 respectively).

The mean values of pairwise LD for neutral and outlier loci classes, across species, were not significantly different (mean difference = 0.026, *t*_23_ = 1.591, *p* = 0.125). Overall outlier loci subsets generally had lower mean pairwise LD within species than neutral loci subsets (0.077 *vs*. 0.105). The range in mean pairwise LD values across species was greater for the outlier loci class (0 – 0.333 *vs*. 0.077 – 0.157), however variance around population means were qualitatively similar for both outlier and neutral loci classes (Additional file 6: Table S5, Additional file 7: Figure S2).

### Association between genomic outlier loci and putative adaptive traits

Across all lake flocks, a total of 43 significant associations between putative adaptive traits and AFLP markers were found (Table 3, Additional file 8: Table S6). The divergent trait with the largest numbers of significant associations with AFLP alleles, across all lake flocks, was gill-raker number (19 loci), followed by LM PC 1 (jaw widths) (eight), spawning depth (six), spawning date (four), GM PC 2 (head length), LM PCs 2 (eye and head size) and 3 (skull widths)(each two). No significant associations were found for GM PCs 3 (head position) and 4 (caudal peduncle length). Within lake flocks the largest number of significant associations were found in Lake Thun (17), followed by L. Lucerne (nine), L. Walen (six), L. Brienz (five), L. Zuerich (four), L. Constance (two) and none in lakes Neuchâtel, Biel and Maggiore.

**Table 3:** Number of AFLP loci revealing a significant association with each putative adaptive trait, for each species flock

			**# Significant Loci**					
**Species Flock**	**GR**	**LM PC 1**	**LM PC 2**	**LM PC 3**	**GM PC 2**	**Date**	**Depth**	**Total**
Neuchâtel	0/ 0/ 0	0/ 0/ 0	0/ 0/ 0	0/ 0/ 0	0/ 0/ 0	0/ 0/ 0	0/ 0/ 0	0
Biel	0/ 0/ 0	n/a	n/a	n/a	0/ 0/ 0	0/ 0/ 0	0/ 0/ 0	0
Thun	2/ 4/ 4	3/ 5/ 6	0/ 0/ 0	1/ 2/ 2	0/ 0/ 1	0/ 0/3	0/ 0/ 1	17
Brienz	0/ 2/ 4	0/ 0/ 0	0/ 0/ 0	0/ 0/ 0	0/ 0/ 0	0/ 0/ 1	0/ 0/ 0	5
Lucerne	1/ 2/ 5	0/ 0/ 0	0/ 0/ 1	0/ 0/ 0	0/ 0/ 0	0/ 0/ 0	2/ 3/ 3	9
Constance	0/ 0/ 1	0/ 0/ 1	0/ 0/ 0	0/ 0/ 0	0/ 0/ 0	0/ 0/ 0	0/ 0/ 0	2
Walen	0/ 1/ 1	0/ 1/ 1	0/ 0/ 1	0/ 0/ 0	0/ 0/ 1	0/ 0/ 0	0/ 0/ 2	6
Zuerich	0/ 4/ 4	0/ 0/ 0	0/ 0/ 0	0/ 0/ 0	0/ 0/ 0	0/ 0/ 0	0/ 0/ 0	4
Maggiore	0/ 0/ 0	0/ 0/ 0	0/ 0/ 0	0/ 0/ 0	0/ 0/ 0	0/ 0/ 0	0/ 0/ 0	0
Total	19	8	2	2	2	4	6	43

Numbers in adaptive trait columns refer to the total number of loci found at each level of significance using the *G*–statistic (*p* = < 0.01/ *p* = < 0.05/ *p* = < 0.1). Adaptive traits used: gill-raker number (GR), linear morphometric PCA scores (LM PC 1, PC 2, PC 3), geometric morphometric PCA scores (GM PC 2), spawning date and spawning depth. n/a = analyses that could not be carried out due to missing data. GM PC 3, PC 4 are not represented as no significant associations were found for these variables.

Four loci showed significant parallel trends of association with adaptive traits in multiple lake flocks. As in the candidate loci screen, CAGC-235 again showed parallel allelic trends; the presence allele was significantly associated in Lakes Lucerne and Brienz with lower gill-raker numbers. The CTAC-99 presence allele was significantly associated with increased gill-raker numbers in lakes Brienz and Thun. The presence allele of CGAG-186 was found in individuals with high gill-raker numbers in L. Brienz and individuals with more negative LM PC 3 scores (reduced skull widths) in L. Thun. The presence allele of CTAG-173 had a significant association with earlier spawning dates in L. Thun and with lower gill-raker numbers in L. Lucerne.

The total number of trait-associated loci found using MatSAM, also detected as F_ST_ outlier loci was higher for Dfdist (26) than for BayeScan analyses (11). However the average percentage of loci that were both outlier and trait-associated, were very similar between the sets of loci identified with Dfdist (22%), and BayeScan (21%)(Additional file 9: Table S7). Overall the lake flock with the highest number of selection candidate loci significantly associated with adaptive trait values was Thun (12 loci). Lakes Brienz, Lucerne and Zuerich came next highest, each having 4 loci, with Walen having two such loci, the remaining species flocks having none.

### Tests of association between heterogeneous genomic divergence and eutrophication

**Figure 4:**
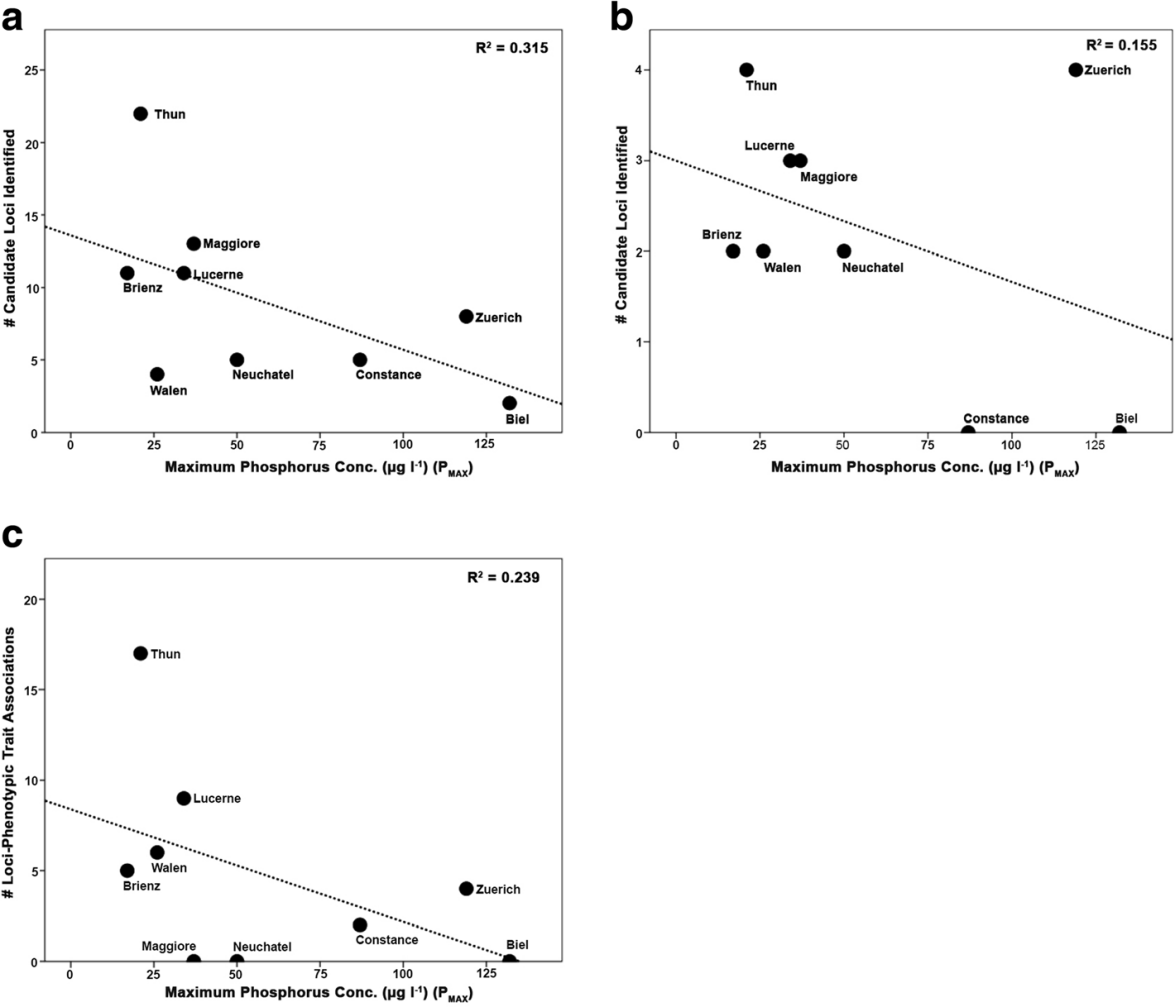
**The effects of historical phosphorus concentration (P**_**max**_**) on the number of candidate loci identified.** Linear regressions of the effects of historical phosphorus concentration (P_max_) on the number of candidate loci identified within lake flocks in (**a**) Dfdist analyses, (**b**) BayeScan analyses and (**c**) the number of significant trait-loci associations in MatSAM.

The number of selection candidate and trait-associated loci identified at the species flock level through the three different analyses methods were significantly positively correlated in two of the three comparisons: Dfdist versus BayeScan (*r*_8_ = 0.717, *P* < 0.05) and Dfdist versus MatSAM (*r*_8_ = 0.763, *P* < 0.05). Results from BayeScan and MatSAM also tended to be positively correlated (*r*_8_ = 0.570, *P* = 0.1). All linear regressions of the effects of P_max_ on the number of selection candidate loci and significant trait-locus associations showed negative, albeit marginally non-significant relationships (Dfdist global comparisons *r*^2^ = 0.315, *P* = 0.058; BayeScan global comparisons *r*^2^ =0.155, *P* = 0.148; MatSAM*r*^2^ = 0.239, *P* = 0.09; all *P* values one-tailed: Figure 4). The regression effect of Min. O_2_ on the number of selection candidate loci and trait-locus associations was positive, but weaker than that of P_max_ and non-significant (Dfdist global comparisons *r*^2^ = 0.263, *P* = 0.097; BayeScan global comparisons *r*^2^ =0.009, *P* = 0.413; MatSAM*r*^2^ = 0.236, *P* = 0.111; Additional file 10: Figure S3).

### Tests of association between neutral genomic divergence and eutrophication

**Figure 5:**
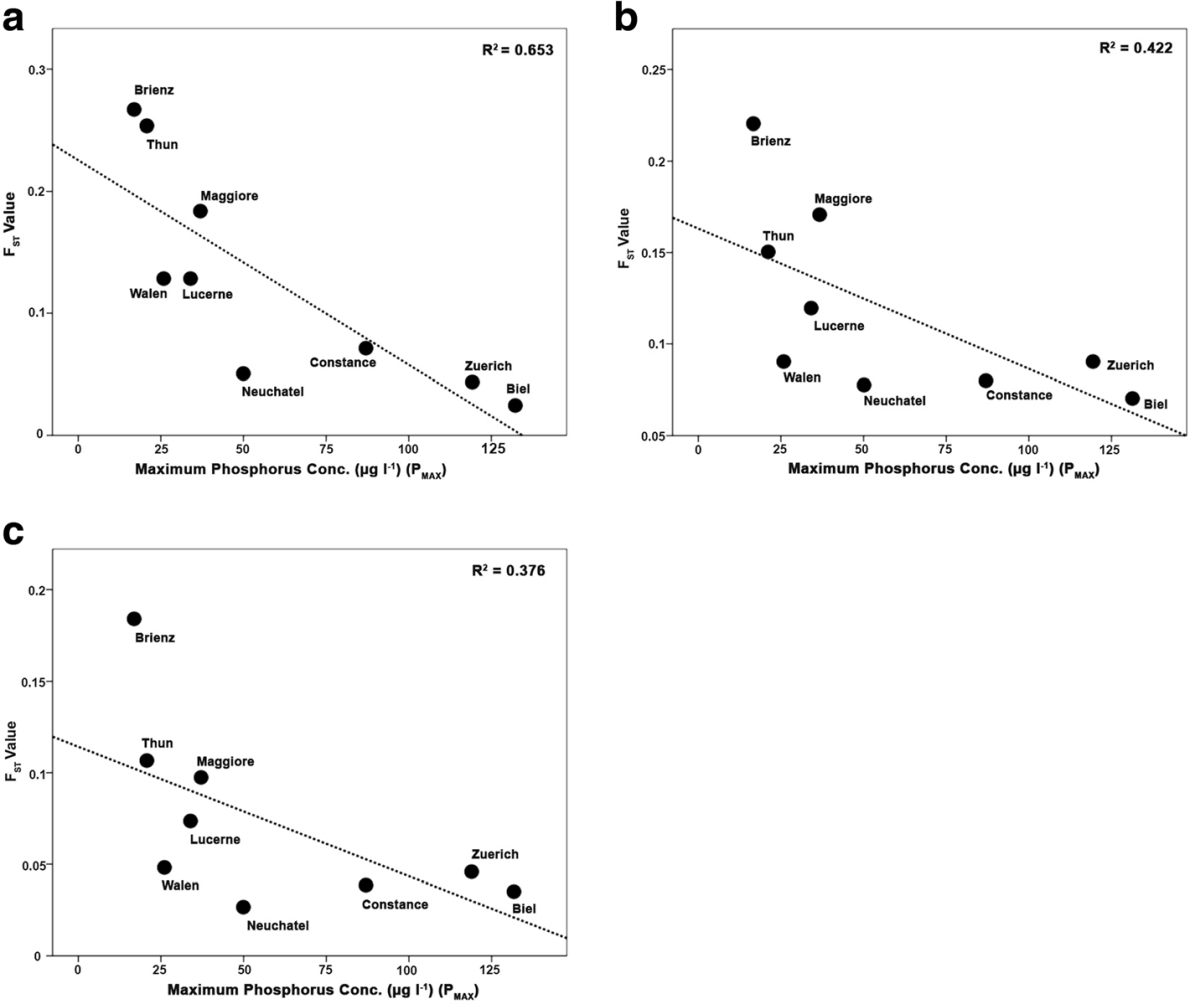
**The effects of historical phosphorus concentration (P**_**max**_**) on overall lake flock genetic differentiation.** Linear regressions of the effects of historical phosphorus concentration (P_max_) on within-lake genetic differentiation among species in different sets of genetic marker loci: (**a**) neutral microsatellite loci, (**b**) all AFLP loci, (**c**) neutral AFLP loci.

In Linear regressions, P_max_ values were strongly negatively correlated with current lake flock-wide global F_ST_ for neutral microsatellites, all AFLP markers and the neutral AFLP subsets (*r*^2^ = 0.38-0.65, *P* = 0.008-0.079; Table 4, Figure 5). Levels of Min. O_2_ were significantly positively correlated with lake flock-wide global F_ST_ and explained even more variation than P_max_ (*r*^2^ = 0.56-0.76, *P* = 0.005-0.033; Additional file 11: Figure S4). Estimates of F_ST_ from all three datasets were highly positively correlated across lakes (*r* = 0.906-0.975, d.f. = 8, *P* <0.001).

**Table 4:** **Maximum within-lake F**_**ST **_**values among sympatric species derived from different genetic markers**

**Species Flock**	**AFLP**		**Microsatellite**
	**All**	**Neutral**	
Neuchâtel	0.077	0.028	0.05*
Biel	0.073	0.035	0.024†
Thun	0.153	0.107	0.252†
Brienz	0.229	0.185	0.269†
Lucerne	0.123	0.074	0.13*
Constance	0.087	0.039	0.071*
Walen	0.095	0.049	0.13*
Zuerich	0.095	0.045	0.044*
Maggiore	0.173	0.097	0.182‡

Comparison of the maximum within-lake pairwise F_ST_ values for each lake flock. All = F_ST_ value derived using all genotyped AFLP loci. Neutral = F_ST_ value derived following removal of all AFLP selection candidate loci. Data taken from: * Vonlanthen *et al*. (2012), † Bittner (2009), ‡ Hudson *et al*. (unpublished observations).

## Discussion

During early stages of ecological speciation, gene flow between incipient species in sympatry/parapatry is expected to be restricted mainly at loci underlying adaptive traits and associated physically linked loci: resulting in increased levels of genetic differentiation in these regions of the genome, relative to unlinked loci evolving under neutrality. In this study we find candidates for loci evolving under disruptive natural selection in each of the examined lake species flocks of the Alpine *Coregonus* adaptive radiation. We also find significant associations between many of these candidate loci and putative adaptive traits, supporting the action of disruptive natural selection in the origin and coexistence of species within this radiation. Some of the lakes have recently undergone anthropogenic homogenization of whitefish habitat through eutrophication, and all analysis methods show negative trends in the number of disruptive selection candidate loci (Dfdist, BayeScan) and the number of significant loci-adaptive trait associations (MatSAM) found among coexisting species, in relation to levels of historical eutrophication within the lakes the whitefish species flocks inhabit. This is congruent with patterns of genetic differentiation shown by neutral microsatellite and AFLP loci in the same species flocks, indicating the weakening of reproductive isolation during eutrophication. We hypothesize that the pattern of reduced numbers of candidate loci found in species flocks that have experienced more severe anthropogenic eutrophication, could be due to two distinct but non-exclusive genomic processes brought about by increased interspecific hybridization and a concomitant increase in interspecific recombination. In the first, increased interspecific recombination erodes genomic islands of elevated genetic differentiation surrounding the target loci of disruptive natural selection, akin to a reversal of divergence hitchhiking, reducing the likelihood that AFLP restriction sites will fall within these regions. In the second, large chromosomal segments, including intact genomic islands of divergence, pass between the genomes of hybridizing species, equalizing allele frequencies and reducing heightened F_ST_ levels associated with genomic islands and therefore reducing the power to detect outlier candidate loci within those segments. Our results presented here provide the first genomic evidence that eutrophication may lead to the relaxation of divergent selection that in turn permits increased levels of interspecific, introgressive hybridization, driving speciation reversal.

### Evidence for the role of disruptive natural selection in shaping intralacustrine diversity

Previous phylogenetic and population genetic analyses have shown that the Alpine *Coregonus* radiation is monophyletic and that its constituent lake flocks form a nested series of independent radiations that arose rapidly, *in-situ*, within the last 15,000 years [25]. During the course of an adaptive radiation, disruptive natural and sexual selection regimes experienced by species in contrasting environments drive adaptive divergence and speciation [59]. Given the co-occurrence of multiple, ecologically divergent species within the Alpine lake flocks and the overall youth of the radiation, heterogeneous genomic divergence between divergent species is expected [14]. Natural selection against hybrids with trait values or trait combinations maladaptive in either parental environment will maintain stronger differentiation in the face of gene flow in genomic islands of elevated interspecific genetic differentiation surrounding loci under disruptive selection than in regions invisible to selection [12]. We find candidate loci displaying unusually strong patterns of genetic differentiation between paired coexisting species in each of the species flock studied.

Given the recent, common origin of the Alpine radiation, parallel trends in candidate loci between similar ecotypes in different lake flocks could be expected. Shared standing genetic variation and similar levels of genetic co-variation among traits increases the likelihood that the same loci and alleles are involved in the evolution of equivalent phenotypes in different lake flocks [60]. We find support for this notion, albeit the number of loci that are repeatedly detected as outliers and/or associated with putative adaptive traits is moderate. Also there is no obvious increase in the phylogenetic grouping of species by ecotype in trees constructed solely from identified outlier loci, and matrices coding for ecotype designation in Mantel tests explained non-significant levels of variation in interspecific genetic distances generated solely from identified outlier loci: though still more than the variation explained in tests using neutral AFLP loci. The moderate number of parallel allelic trends likely reflects that many of the candidate loci we identified, are linked with varying degrees of physical proximity to loci under disruptive natural selection, rather than being the target loci of disruptive selection themselves [13,61]. During speciation within the independent whitefish species flocks, the action of recombination, eroding blocks of linkage disequilibrium along the genome, and the time since origination of the beneficial alleles, could mean that different genetic backgrounds containing different hitchhiker AFLP outlier loci are driven to, or near to fixation in similar ecotypes, among species flocks: despite the true target locus of selection, that hitchhiking loci are physically linked too, being identical in each paralleled speciation event [60]. Therefore parallel trends in candidate loci between similar ecotypes would likely be restricted to hitchhiker loci very tightly linked to the actual loci under selection and/or parallel population divergences of recent, shared evolutionary origins.

From our study, locus CAGC-235 showed parallel allelic trends in more than two lake systems. The presence allele for this locus has a reduced prevalence in lower gill-raker ecotypes in lakes Maggiore, Thun (*C*. *fatioi* only), Zuerich, Lucerne and Brienz (*C*. *sp*. “balchen” only). Therefore there is the potential that the alternative allele for CAGC-235 is tightly linked to the actual target locus for natural selection. Five of the remaining loci support the role of recombination in breaking down the physical linkage between homologous candidate loci and the target of selection over evolutionary time: the parallel trends in these loci being shared only between the most recently separated lake flocks. Three loci were found in both lakes Thun and Brienz (CGAG-186, TGTG-183, CTAC-99), which form part of the same monophyletic super-lake radiation and given the geographical proximity of the lakes, were likely the last of the studied species flocks to separate. CGAG-355 and TGTG-271 show parallel allelic trends between introduced L. Maggiore species and their likely source populations in either lakes Lucerne and Constance [25].

### Anthropogenic perturbations and heterogeneous genomic divergence

The genomic extent of levels of increased genetic differentiation around the target loci for disruptive natural selection will depend on the balance between the strength of selection and gene flow [12,13]. Two distinct, but to some degree complimentary, mechanisms have been proposed to describe how genetic differentiation spreads through the genomes of species diverging under speciation-with-gene-flow scenarios. Under divergence hitchhiking (DH), strong disruptive selection acting on a locus reduces interspecific recombination over relatively restricted areas of the genome in physical linkage with the selective locus. In this way beneficial alleles under weaker selection can be recruited for adaptive divergence through their “uplift” onto genomic islands of divergence surrounding more strongly selected loci. This reduces the chances of their loss to interspecific recombination and facilitates the growth of genomic islands of divergence [13,62]. Under genome hitchhiking (GH), disruptive selection reduces the average effective migration across the entire genome, thereby facilitating differentiation throughout the genome irrespective of levels of physical linkage to selective loci [63,64]. Feder, Egan & Nosil [64] describe a four-phase model of speciation-with-gene-flow. In this model, DH is most important in facilitating genomic differentiation at early to intermediate stages of speciation (phase 2). At later stages, as more loci are recruited by disruptive natural selection, GH will become the dominant mechanism driving genomic differentiation (phase 3). The relatively recent origin of the species flocks of the Alpine *Coregonus* radiation (<15,000 years) suggests that DH could play an important role in the divergence seen between species. Under DH, genomic islands of divergence around selective loci are expected to increase in size as speciation progresses. This in turn would increase the likelihood that individual AFLP loci will fall within these areas of higher genetic differentiation and be classed as outlier candidate loci. Therefore as genomic divergence progresses under DH, the proportion of outlier candidate loci amongst a random genomic sample of AFLP loci should increase: relative to a similar genome scan conducted at an earlier time point in the speciation process of the same species pair [12,13]. This prediction depends on the existence of relatively extensive genomic regions of low interspecific recombination around selected loci. In dwarf/normal species pairs in the closely related *Coregonus clupeaformis* species complex, effective migration was found to be reduced for relatively large distances around selective loci (≈ 5 cM) and that the extent of phenotypic divergence between coexisting species pairs was positively correlated with levels of linkage disequilibrium around these loci [48,65]. However, in the species flocks of the Alpine whitefish radiation more genomic research will be needed to characterize the actual target loci of selection and define the effective limits of surrounding islands of divergence, taking into account factors which could potentially enhance (*e*.*g*. epistasis) or constrain (*e*.*g*. selection from standing genetic variation) the size of these genomic areas: before extensive DH can be confirmed [13,63].

In the lakes of the Alpine *Coregonus* radiation, previous research has shown that historical levels of anthropogenic nutrient enrichment negatively correlated with contemporary interspecific genetic differentiation at neutral markers, and positively correlated with the overall loss of species and functional phenotypic diversity. Direct comparisons of neutral genetic differentiation from samples taken from the same species flocks, pre- and post- (large-scale) eutrophication, corroborate this pattern, with strong declines found in a highly eutrophied lake relative to more lightly polluted lakes, despite similar levels of genetic differentiation occurring in these species flocks prior to the peak of eutrophication [8]. Our present study corroborates these results, with genetic differentiation at selectively neutral subsets of AFLP loci also being strongly negatively correlated with levels of anthropogenic nutrient enrichment. Focusing on areas of the genome evolving under the influence of disruptive natural selection, we hypothesized that the numbers of candidate loci identified within a lake flock would be also negatively correlated with each lake’s historical maximum level of eutrophication. As the lake habitats become increasingly homogeneous during eutrophication, disruptive selection between habitats would wane, reducing the efficacy of barriers to interspecific gene flow and resulting in increased interspecific recombination that would, in turn, either reduce the size of genomic islands of divergence, reversing divergence hitchhiking or result in the transfer of entire chromosomal segments containing intact genomic islands between the genomes of hybridizing species, equalizing allele frequencies at potential F_ST_ outlier loci. However, whilst we find that numbers of candidate loci in lakes with less strong eutrophication histories were indeed high (lakes Thun, Brienz, Lucerne) and were lowest in the most eutrophic lake (Biel), the relationship with phosphorus was marginally non-significant. This was due to certain lakes having fewer candidate loci (Neuchâtel, Walen) and others lakes having more (Zuerich), than would be predicted given their past levels of eutrophication.

Variation in the levels of ecological divergence between coexisting species, among species flocks, prior to environmental perturbations could potentially affect the relative numbers of candidate loci found among lake flocks today. If ecological divergence and therefore development of RI was impeded by high levels of gene flow, weaker disruptive selection and/or temporal fluctuations in selection regimes, this would affect the number and extent of genomic islands of divergence that occur between coexisting species naturally and how susceptible they are to environmental disturbance when it occurs and subsequently the extent of speciation reversal, relative to similarly aged species flocks from more ecologically divergent/stable lake environments [4,66]. The two pairs of coexisting species from recently connected lakes: Walen and Zuerich, and Biel and Neuchâtel, represent two distinct super-lake clades within the overall Alpine radiation [25]. Lakes Zuerich and Biel have a similar history of high eutrophication (P_max_ = 119 μg/l and 132 μg l^-1^ respectively), L. Neuchâtel moderate (50 μg/l) and L. Walen low (26 μg/l). Using gill raker number as a surrogate for ecological diversity among coexisting species, L. Zuerich had a greater pre-eutrophication difference in average gill-raker number between species (26.7 & 35.2) than lakes Biel (27.4 & 33.2) or Neuchâtel (27.5 & 32.2), similar to L. Walen (25.8 & 35.4)[8,29]. Following heavy eutrophication, despite evidence of increased neutral gene flow and low microsatellite F_ST_ values similar to Biel and Neuchâtel [8], a higher number of candidate loci was found and relatively high phenotypic differentiation has been maintained between L. Zuerich species, despite much more severe levels of pollution relative to Neuchâtel. Additional support for this hypothesis may come from the closely related, North American *C*. *clupeaformis*-complex. The coexisting *Coregonus* species of Indian pond show high levels of phenotypic differentiation and the largest number of AFLP candidate loci yet found in the *C*. *clupeaformis* radiation, yet have relatively low neutral genetic differentiation [18,67]. Intriguingly, Indian Pond has phosphorus concentrations three times higher than other studied North American *Coregonus* lakes and the highest post-summer decreases in oxygen with depth, suggesting this lake and its endemic *Coregonus* species may have experienced eutrophication in its recent past [68].

In pristine environments, as the number of species within a radiation increases so should the number of selection candidate loci and trait associated loci identified, as the number of axes of divergent adaptation among coexisting species increases [65]. In the Lake Thun species flock, five species coexist and the highest numbers of candidate loci/trait associated loci found in this study were identified within this species flock. In contrast, the similarly species-rich, yet heavily polluted L. Constance species flock had some of the lowest numbers of candidate loci/trait associated loci identified in the Alpine radiation. L. Constance previously held up to six coexisting species, but following eutrophication, the VLGR ecotype, *C*. *gutturosus* went extinct and among the remaining species a large decrease in interspecific neutral genetic differentiation occurred [8]. This may illustrate a potential trend in speciation reversal, in that young lake radiations with higher species diversity are intrinsically more vulnerable, in response to a given level of environmental change, to speciation reversal than less species-rich systems. Within evolutionarily young, species-rich radiations, ecological and reproductive niche breadths may be narrower and more tightly packed compared to related, less diverse radiations, perhaps through partial subdivision of the ancestral niche: potentially making them more sensitive to relaxation of disruptive natural selection regimes. As environmental factors imparting RI begin to break down the increased potential directions of gene flow between multiple coexisting species could in turn exacerbate speciation reversal. This hypothesis could help to explain rapid collapses in diversity within other young radiations such as the Lake Victoria cichlids [5] and Great Lake ciscoes [9].

The strategy we have adopted for the present study was to investigate all possible Alpine whitefish species flocks, including most of the endemic species present: each species flock comprising a distinct data point along a spectrum of anthropogenic habitat perturbation from low to severe. Overall, the limited number of individuals per species included in our analyses certainly places limitations on the power to identify, among the set of loci sampled, all those that might be evolving under disruptive selection between coexisting species. Also, whilst mean values of linkage disequilibrium were not higher among identified outlier loci than among neutral loci, suggesting that detected outliers have a rather dispersed distribution in the genomes of Alpine whitefish species, the true number of separate linkage groups that our candidate loci comprise remains unknown. Additionally, we cannot rule out entirely that another, unidentified ecological factor, covarying with the extent of eutrophication, may be a stronger driver of the pattern seen in the number of outlier loci identified amongst Swiss whitefish species flocks. In this regard, our study stands as a necessary first step, allowing future studies to home in on the actual target loci of disruptive selection and their function, to uncover the genomic architecture of the phenotypic traits they affect and to measure the fitness effects of alleles at these loci within the alternate niches of the different species, and how eutrophication may affect these. Given the number and diversity of species flocks covered by our sampling scheme, which explicitly takes into account the hierarchical structuring of genetic variation within the Alpine whitefish radiation; we have the power to look at the relative numbers of candidate loci identified and formulate hypotheses as to the factors influencing variation in this number. Therefore despite the fact that while some of the individual candidate loci identified in this study, may have elevated genetic differentiation through processes other than contemporary disruptive natural selection, such as historical selective sweeps [69], demography [70] or their location in chromosomal regions with reduced recombination [21]; the recent, common origin of the species flocks of the Alpine whitefish radiation means that these potential biases are very unlikely to lead to the trends in candidate loci we find across lake flock data sets. Furthermore by using two different F_ST_ outlier detection methods, alongside logistic regressions, combining population-based and individual-based approaches respectively, not only can we identify adaptive traits important in niche differentiation and the loci potentially underpinning them, but also that the high congruence in the results produced by these different methodologies suggests that the overall patterns in candidate loci revealed by our study are real.

## Conclusions

Within each of the postglacial species flocks of the Alpine whitefish radiation we find evidence for the action of disruptive natural selection in the shaping of within-lake species diversity through the identification of F_ST_ outlier loci and/or significant adaptive trait/candidate loci associations. We also find, similar to the extent of interspecific genetic differentiation at neutral loci, a negative correlation between the number of identified F_ST_-outlier loci identified and significant adaptive trait/candidate loci associations, and historical levels of anthropogenic nutrient enrichment within each lake. We hypothesize that the habitat disruption caused by eutrophication weakens disruptive natural selection regimes resulting in increased gene flow between species. The resulting interspecific recombination either breaks down genomic islands of heightened genetic differentiation surrounding the target loci of disruptive natural selection or entire chromosomal segments, including intact genomic islands, pass between species’ genomes, homogenizing allele frequencies. Our study presents the first genomic evidence that speciation reversal is associated with the relaxation of disruptive selection: contributing to our understanding of the mechanisms that control this important driver of biodiversity loss.

## Competing interests

The authors declare that they have no competing interests.

## Authors’ contributions

AGH wrote the paper, contributed to conception and design of the study, sampled fish, generated adaptive trait and genetic data, and carried out most of the subsequent analyses. PV sampled fish, generated environmental lake data and contributed to analyses and writing. EB contributed to analyses and writing. OS conceived and designed the project, supervised the project, and contributed to data analyses and writing. All authors read and approved the final manuscript.

## Supplementary Material

Additional file 1: Table S1 Individual phenotypic trait values. Click here for file

Additional file 2: Table S2 AFLP primer pair information. Click here for file

Additional file 3: Table S3 Identity of AFLP loci classified as F_ST_ outliers following Dfdist analyses. Click here for file

Additional file 4: Table S4 Identity of AFLP loci classified as F_ST_ outliers following BayeScan analyses. Click here for file

Additional file 5: Figure S1 Population-based NJ consensus trees created using different subsets of AFLP loci. Click here for file

Additional file 6: Table S5 Mean pairwise LD values from neutral and outlier loci classes for each whitefish species. Click here for file

Additional file 7: Figure S2 Distribution of pairwise linkage disequilibrium values for neutral and outlier loci classes within each whitefish species. Click here for file

Additional file 8: Table S6 Identity of AFLP loci with significant associations with putative adaptive phenotypic traits identified by matSAM. Click here for file

Additional file 9: Table S7 Congruence between Global dataset evidence for disruptive selection and association with adaptive traits within species flocks. Click here for file

Additional file 10: Figure S3 Linear regressions of the effects of minimum lake oxygen concentration at depth (Min. O_2_) on the number of candidate loci identified within lake flocks in (**a**) Dfdist analyses, (**b**) BayeScan analyses and (**c**) the number of significant trait-loci associations in MatSAM. Click here for file

Additional file 11: Figure S4 Linear regressions of the effects of minimum oxygen concentration at maximum depth (Min. O_2_) on genetic differentiation in different sets of loci: (**a**) neutral microsatellite loci, (**b**) all AFLP loci (**c**) ‘neutral’ AFLP loci. Click here for file
