# Supplementary material for: Genomic signatures of relaxed disruptive selection associated with speciation reversal in whitefish
Source: BMC Evol Biol. 2013 May 30;13:108. doi: 10.1186/1471-2148-13-108 (PMC3685556; doi:10.1186/1471-2148-13-108)

**Figure S1.** Population-based NJ consensus trees for different subsets of AFLP loci used in F_ST_ outlier analysis: a) all 835 AFLP loci used in this study, b) 739 “neutral” AFLP loci, c) 96 loci indentified as selection candidate loci. Numbers in bold refer to % bootstrap support (1000 replicates). Branch colours refer to the radiation of origin: Neuchâtel-Biel (pink), Thun-Brienz (orange), Lucerne (blue), Walen-Zuerich (green), Constance (red) and Maggiore (purple).


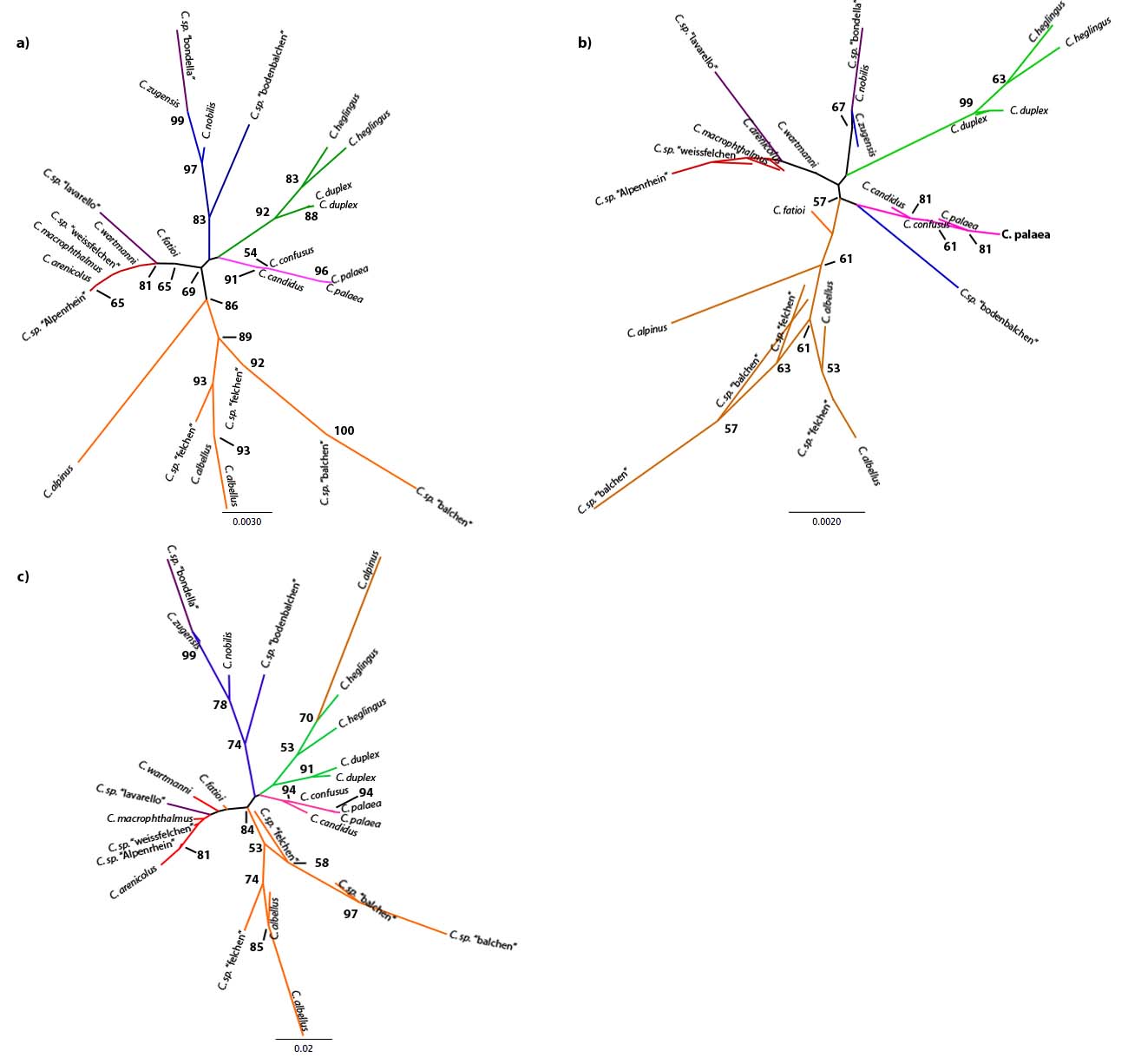

Supplement: Additional file 5: Figure S1 — Population-based NJ consensus trees created using different subsets of AFLP loci. [file 1471-2148-13-108-S5.doc]
