# Supplementary material for: Genomic signatures of relaxed disruptive selection associated with speciation reversal in whitefish
Source: BMC Evol Biol. 2013 May 30;13:108. doi: 10.1186/1471-2148-13-108 (PMC3685556; doi:10.1186/1471-2148-13-108)

**Figure S2.** Pairwise linkage disequilibrium among different AFLP loci classes within whitefish species. Filled diamonds indicate mean pairwise linkage disequilibrium ($\bar{r}_{d}$) for outlier loci (S) subsets (red) and neutral loci (N) subsets (blue) per species. Green error bars show ± 1 standard deviation around the mean pairwise LD. Lower case letters refer to the constituent species within each species flock: Neuchâtel (a) *C*. *palea*, (b) *C*. *candidus*; Biel (c) *C*. *palaea*, (d) *C*. *confusus*; Thun (e) *C*. *fatioi*, (f) *C*. *sp*. “balchen”, (g) *C*. *albellus*, (h) *C*. *alpinus*, (i) *C*. *sp*. “felchen”; Brienz (j) *C*. *sp*. “balchen”, (k) *C*. *sp*. “felchen”, (l) *C*. *albellus*); Lucerne (m) *C*. *zugensis* (n) *C*. *sp*. “bodenbalchen”, (o) *C*. *nobilis*; Constance (p) *C*. *macrophthalmus*, (q) *C*. *wartmanni*, (r) *C*. *sp*. “Alpenrhein”, (s) *C*. *sp*. “weissfelchen”, (t) *C*. *arenicolus*; Walen (u) *C*. *duplex*, (v) *C*. *heglingus*; Zuerich (w) *C*. *heglingus*, (x) *C*. *duplex*; Maggiore (y) *C*. *sp*. “lavarello”, (z) *C*. *sp*. “bondella”.


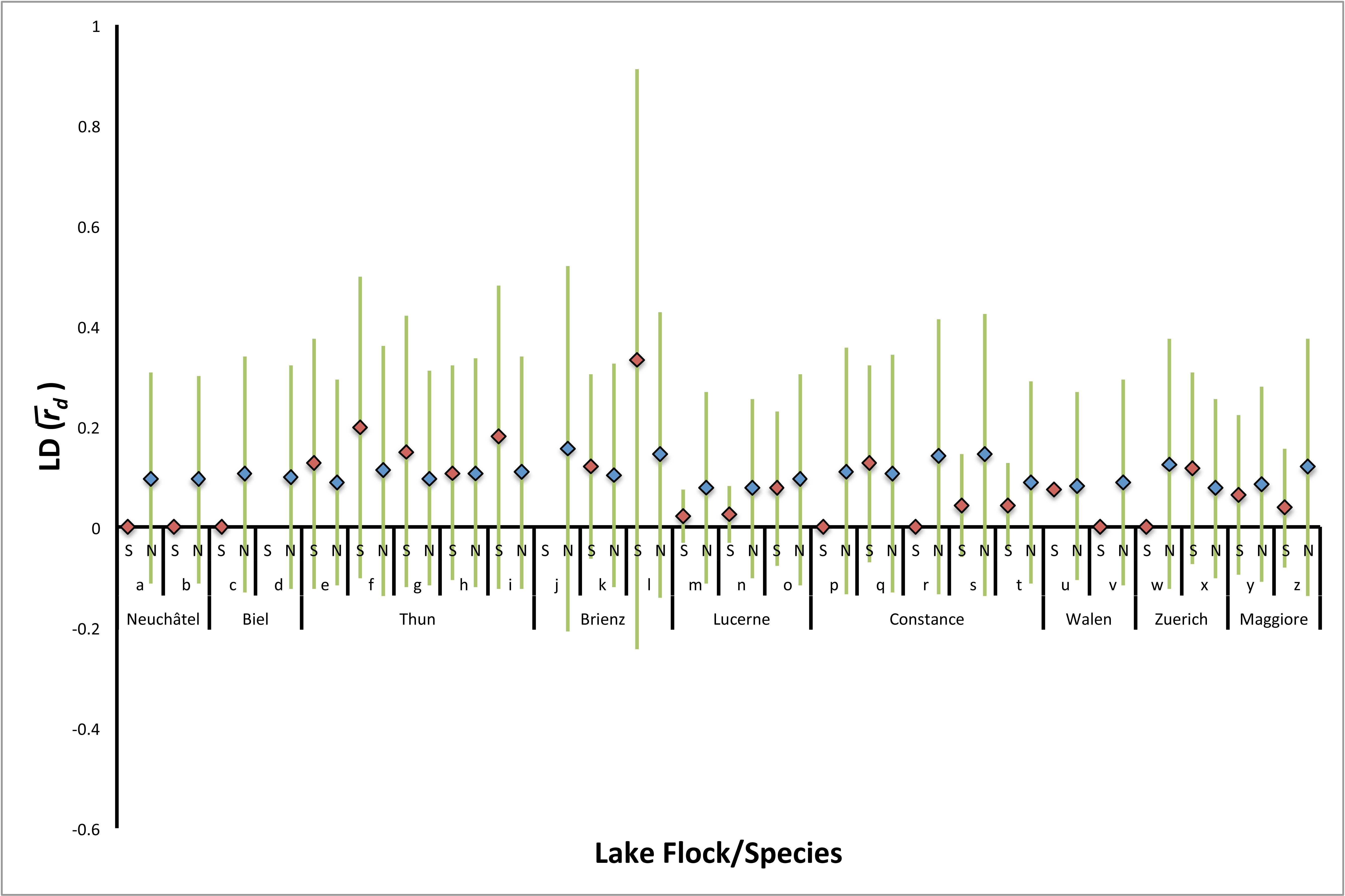

Supplement: Additional file 7: Figure S2 — Distribution of pairwise linkage disequilibrium values for neutral and outlier loci classes within each whitefish species. [file 1471-2148-13-108-S7.docx]
