# Supplementary material for: Genomic signatures of relaxed disruptive selection associated with speciation reversal in whitefish
Source: BMC Evol Biol. 2013 May 30;13:108. doi: 10.1186/1471-2148-13-108 (PMC3685556; doi:10.1186/1471-2148-13-108)

**Figure S3.** Linear regressions of the effects of minimum oxygen concentration at maximum depth (Min. O_2_) on genetic differentiation in different sets of loci: (a) neutral microsatellite loci, (b) all AFLP loci (c) ‘neutral’ AFLP loci.


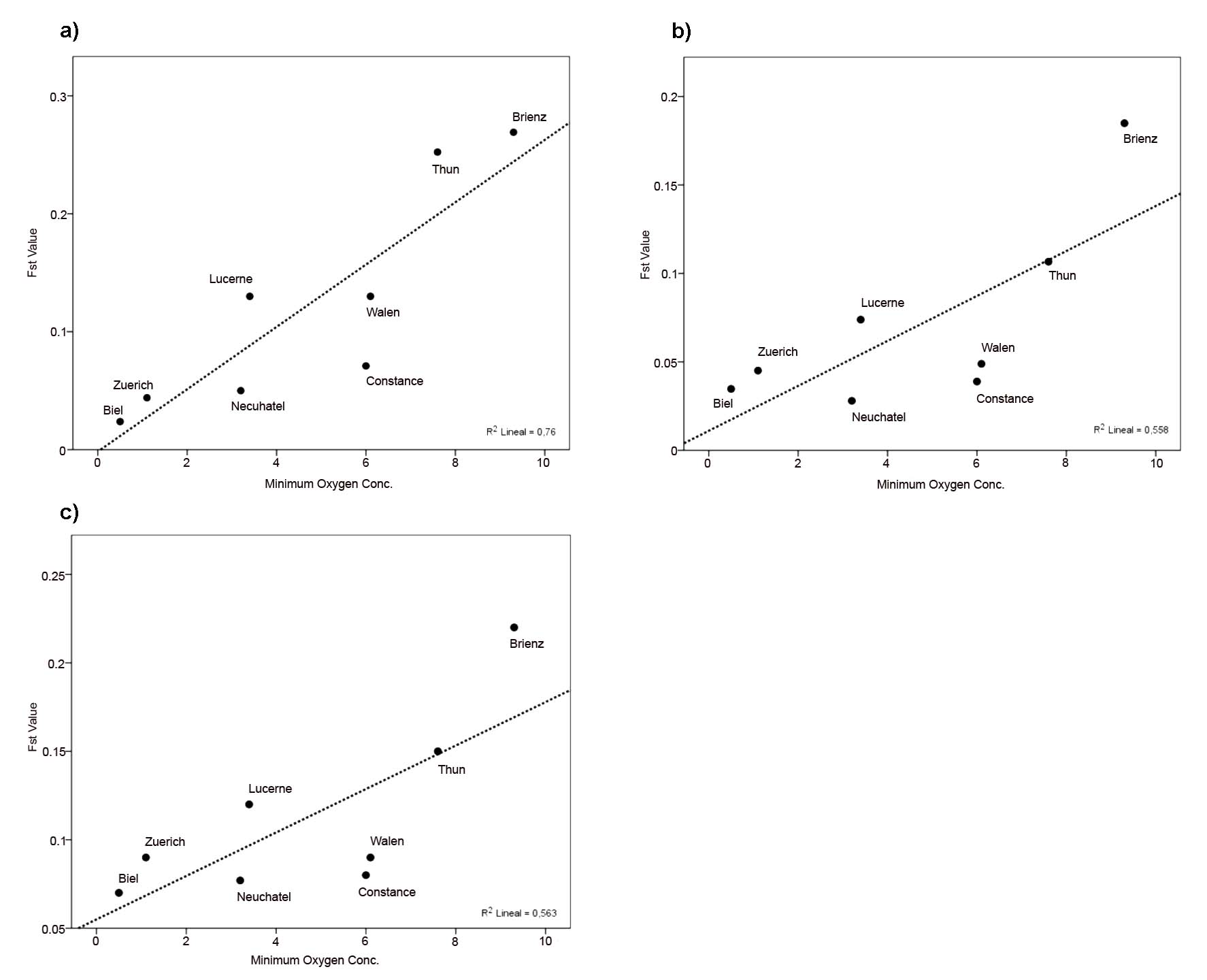

Supplement: Additional file 10: Figure S3 — Linear regressions of the effects of minimum lake oxygen concentration at depth (Min. O2) on the number of candidate loci identified within lake flocks in (a) Dfdist analyses, (b) BayeScan analyses and (c) the number of significant trait-loci associations in MatSAM. [file 1471-2148-13-108-S10.doc]
