# Supplementary material for: Genomic signatures of relaxed disruptive selection associated with speciation reversal in whitefish
Source: BMC Evol Biol. 2013 May 30;13:108. doi: 10.1186/1471-2148-13-108 (PMC3685556; doi:10.1186/1471-2148-13-108)

**Figure S4.** Linear regressions of the effects of minimum lake oxygen concentration at depth (Min. O_2_) on the number of candidate loci identified within lake flocks in (a) Dfdist analyses, (b) BayeScan analyses and (c) the number of significant trait-loci associations in MatSAM


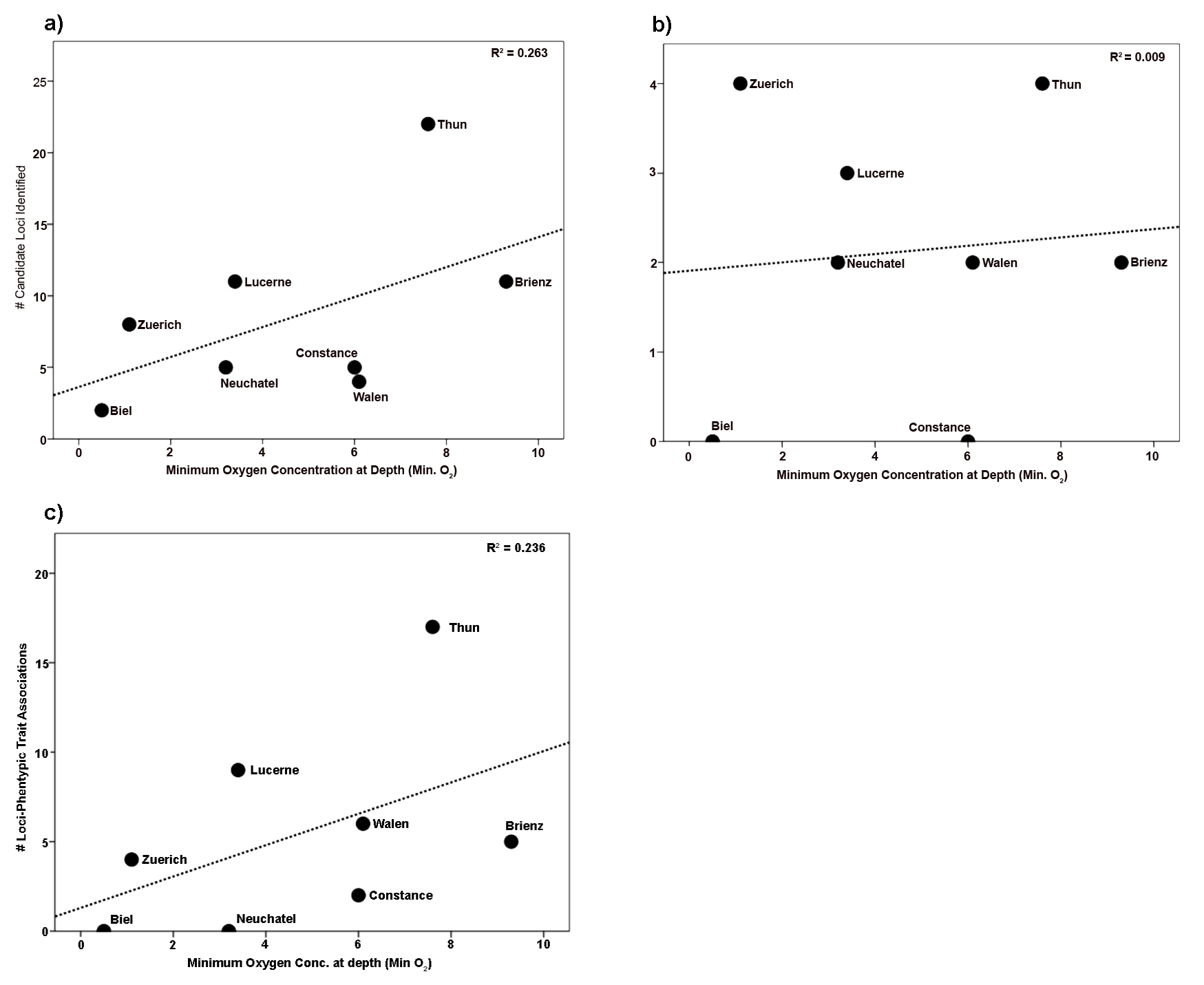

Supplement: Additional file 11: Figure S4 — Linear regressions of the effects of minimum oxygen concentration at maximum depth (Min. O2) on genetic differentiation in different sets of loci: (a) neutral microsatellite loci, (b) all AFLP loci (c) ‘neutral’ AFLP loci. [file 1471-2148-13-108-S11.doc]
